# What People Want: Exercise and Personalized Intervention as Preferred Strategies to Improve Well-Being and Prevent Chronic Diseases

**DOI:** 10.3390/nu17111819

**Published:** 2025-05-27

**Authors:** Nadia Solaro, Eleonora Pagani, Gianluigi Oggionni, Luca Giovanelli, Francesco Capria, Michele Galiano, Marcello Marchese, Stefano Cribellati, Daniela Lucini

**Affiliations:** 1 Department of Statistics and Quantitative Methods, University of Milano-Bicocca, 20126 Milan, Italy; nadia.solaro@unimib.it; 2 Department of Psychology, Catholic University of the Sacred Heart, 20123 Milan, Italy; eleonora.pagani@unicatt.it; 3 Exercise Medicine Unit, Istituto Auxologico Italiano IRCCS, 20135 Milan, Italy; g.oggionni@auxologico.it; 4 BIOMETRA Department, University of Milan, 20129 Milan, Italy; luca.giovanelli@unimi.it; 5 Assidim, 20122 Milan, Italy; francesco.capria@assidim.it (F.C.); michele.galiano@assidim.it (M.G.); marcello.marchese@assidim.it (M.M.); 6 SEGE srl, 20146 Milan, Italy; stefano.cribellati@se-ge.com

**Keywords:** bagging, check-up, classification tree, insurance, lifestyle, nonparametric statistics, stress, well-being, workplace health promotion

## Abstract

**Background/Objectives**: The workplace represents an ideal context for applying policies to foster a healthy lifestyle, guaranteeing advantages both to the individual and the company. Nevertheless, motivation to change one’s lifestyle remains an issue. This study aimed to determine subjects’ most valued intentions toward lifestyle changes and the target actions to improve lifestyles that they would be willing to invest in economically, information which might help design effective intervention programs. **Methods**: Classification trees were applied to 2762 employees/ex-employees (55.09 ± 13.80 years; 1107 females and 1655 males) of several Italian companies who voluntarily filled out an anonymous questionnaire on lifestyles (inquiring about, e.g., exercise, nutrition, smoking, and stress) to unveil specific subject typologies that are more likely associated with, e.g., manifesting a specific intention toward lifestyle changes and choosing the two most popular target actions resulting from the survey. **Results**: The main lifestyle aspect that respondents desired to improve was to become more physically active, and the most preferred tools chosen to improve their lifestyle were the possibility of having a medical specialist consultant to prescribe a tailored lifestyle program and buying a gym/swimming pool membership. **Conclusions**: This observational study might help tailor worksite health promotion and insurance services offered to employees, initiatives that may play an important role in fostering health/well-being and preventing chronic diseases in the more general population, especially in healthy or young subjects who are more prone to change their behavior if immediate benefits are seen instead of only advantages in the future.

## 1. Introduction

Nutrition, exercise, and, in general, a healthy lifestyle nowadays represent a pivotal strategy to manage and reduce the risk of developing many chronic non-communicable diseases, particularly cardiometabolic and oncological ones. Notably, these behavioral strategies are also a substantial tool to foster well-being, assuming a fundamental role not only in approaches to promote future health (prevention) but also in actions to promote current health (well-being) [1].

**Figure 1:**
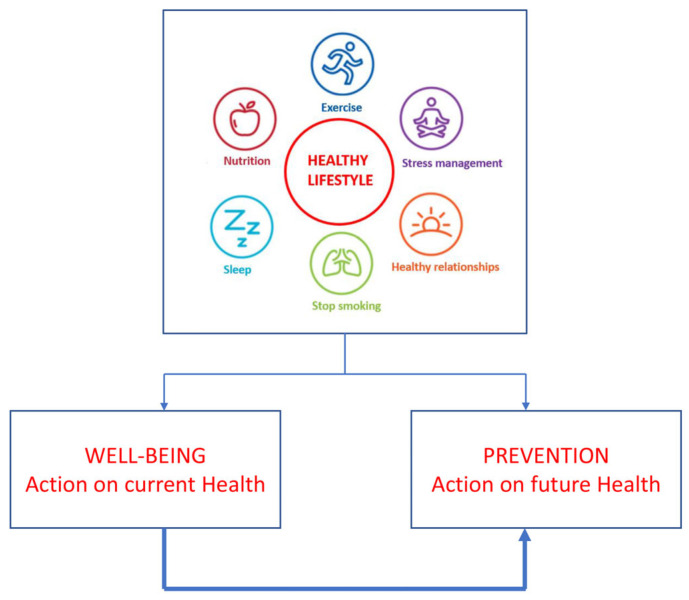
Role of a healthy lifestyle in determining well-being and prevention of chronic diseases.

The availability of tools to encourage well-being in the present might catch the interest of people, especially healthy or young subjects, who are more prone to change behavior if this action could drive an immediate benefit instead of guaranteeing only advantages in the future [2,3,4,5,6]. Motivation to have a healthy diet, be physically active, stop smoking, and find resources to manage stress is hard to obtain, and the possibility of gaining immediate advantages might empower the subject toward a proactive role in improving their lifestyle. Well-being reduction represents, in fact, one of the main concerns of the working population, and strategies to improve it are more and more recommended by institutions such as the World Health Organization and the European Community [1,7]. Concomitantly, adherence to a healthy lifestyle obviously drives the prevention of chronic diseases, guaranteeing a double important result (see Figure 1).

The workplace may represent an ideal context for applying policies to foster a healthy lifestyle, guaranteeing advantages both to the individual and the company, considering the improvement in work performance, productivity, safety, absenteeism reduction, and enhanced corporate climate [6]. Nevertheless, Workplace Health Promotion [8,9] remains an issue [10], and efficacy varies considerably across different approaches to intervention [10,11,12,13,14]. Tailoring intervention to the subjects’ behaviors and needs results in more effective programs [11,12,15,16]. The possibility of assessing subjects’ lifestyles and the knowledge of their needs and preferences might help in designing effective programs, based more on lifestyle modifications (physical activity, nutrition, stress, stopping smoking, etc.) than on the assessment of traditional cardiometabolic risk factors (such as cholesterol levels, blood pressure, etc.) that are frequently within normal range, particularly in young employees. Notably, young subjects are frequently characterized by unhealthy lifestyles, increased stress perception, and reduced well-being [2,3,6].

Another important issue to be considered is the costs of workplace campaigns and interventions to prevent chronic diseases and foster well-being. In most countries, these costs are outside the National Health System coverage and are borne by individual and/or company policies, which frequently consider ad hoc insurance plans.

This study aimed to detect subjects’ most valued intentions toward lifestyle changes and which target actions they would be willing to invest economically to improve their lifestyle. We employed a simple, anonymous online questionnaire [17,18] inquiring about lifestyles and offered it to companies located in the northern part of Italy. A further goal was to verify if an advanced statistical approach based on classification trees [19,20] could be useful in unveiling the characteristics of specific groups of subjects to offer tailored lifestyle intervention programs. We then focused on identifying the key socio-demographic and behavioral variables, collected through the online survey, and the principal associations among their categories that were capable of profiling specific subject typologies with higher probabilities of having particular desires toward their lifestyle changes and having, or not, particular propensities toward making specific target actions to improve their lifestyle.

## 2. Materials and Methods

A group of 4013 employees or ex-employees of several Italian companies randomly filled out, on a voluntary basis, an anonymous questionnaire on lifestyles from January 2024 to May 2024 on the Assidim web page (a non-profit association that provides associated companies and their employees–families financial assistance and support in case of accident, disease, death, and invalidity; https://www.assidim.it (accessed on 16 April 2025)), which considers, since its foundation in 1981, health promotion among workers and associated companies as its mission. The website was accessible by all employees of Italian companies, even if they were not associated with Assidim. To guarantee anonymity, we did not select any specific population among those who could access the Assidim website.

Although anonymous, the questionnaire provided every single participant with a personalized, immediate report based on the information inserted. We recently described [2,3,6] the methodology employed to create this questionnaire, which was validated by statistical analysis [18] and employed in other research whose results had already been published, in particular, the results of an initial survey that we proposed on the web page of Assidim [3,6]. Briefly, the questionnaire was designed to obtain data on lifestyles (diet habits, exercise, smoking, sleep hours, and perception of stress); job categories; participants’ perception of the quality of their personal health, sleep, and job performance; and anthropometric data. Perception of sleep quality and health quality was assessed by providing ordinal self-rated 11-point Likert scales from 0 (“very bad”) to 10 (“very good”) for each measure. Similarly, job performance perception was assessed using ordinal self-rated 11-point Likert scales from 0 (“very bad”) to 10 (“very good”). We also inquired about which lifestyles they would like to improve (only one answer was possible among the following: eating better, being more physically active, managing stress, improving sleep quality, and stopping smoking) and their willingness to invest economically to take actions capable of helping them improve their lifestyles, considering the following items (it was possible to indicate more than one answer): buying healthier food, buying sports equipment and clothing, performing medical tests (check-ups), buying a gym/swimming pool membership, having a medical specialist consultancy for prescribing a tailored lifestyle program, participating in stress management training, and/or programs for quitting smoking. To assess the willingness to invest economically, we considered the following amount ranges: <EUR 100, EUR 100–299.99, EUR 300–499.99, and ≥EUR 500. Special attention was given to the latter question, because it was regarded as a proxy for both the subjects’ willingness to commit to lifestyle changes and their financial readiness.

All the subjects participating voluntarily in the survey inserted their information anonymously in the questionnaire. They were aware of the possible use of the collected data in an anonymized and aggregated form for scientific purposes. This research was part of an ongoing study on the role of questionnaires in assessing lifestyles, approved by the local Ethical Committee (Istituto Auxologico Ethical Committee, code 2023_04_18_14 dated 4 May 2023).

### 2.1. Lifestyle Assessment

As we published in our previous papers (see references [2,3,6,17,18] for details), we assessed lifestyles considering the following items:

- Physical activity (weekly physical activity volume) [21], using the following formulas to assess the moderate-intensity physical activity volume (in MET·minutes/week):
  moderate-intensity = 3.3 × *m*(*BW*) × *d*(*BW*) + 4.0 × *m*(*M*) × *d*(*M*), (1)
  where *BW* denotes brisk walking activity, which was estimated at approximately 3.3 METs/minute; *m*(*BW*) stands for the number of minutes/day of brisk walking performed in a number *d*(*BW*) of days/week; analogously, *M* refers to other moderate-intensity activities, which were estimated at approximately 4.0 METs/minute; and *m*(*M*) is the number of minutes/day of other moderate-intensity activities performed in a number *d*(*M*) of days/week. The vigorous-intensity physical activity volume (in MET·minutes/week) was derived using the following:
  vigorous-intensity = 8.0 × *m*(*V*) × *d*(*V*), (2)
  where *V* stands for vigorous-intensity activities, estimated at approximately 8.0 METs/minute, and *m*(*V*) is the number of minutes/day of vigorous-intensity activities performed in a number *d*(*V*) of days/week. Finally, the total weekly physical activity volume (in MET·minutes/week) was derived by the sum of the two scores (1) and (2):
  total volume = moderate-intensity + vigorous-intensity.(3)
- Nutrition was guessed using the American Heart Association (AHA) diet score [22], adapted to Italian eating habits [17,18].
- Perception of somatic symptoms (short 4SQ), fatigue, and stress were guessed using a self-administered questionnaire [17,18], providing ordinal self-rated Likert scales from 0 (“very good”) to 10 (“very bad”) for each measure. Short 4SQ considers four somatic symptoms; thus, the total score, equal to the sum of the 0–10 scores on the single somatic symptom scales, ranges from 0 to 40.
- Smoking behavior: all subjects who reported having never smoked or had stopped smoking for more than one year were considered non-smokers.

### 2.2. Statistics

A preliminary quality analysis of the collected data was conducted to cut out questionnaires that were not fully completed or questionnaires with non-realistic or incongruent responses from the dataset. A total of *N* = 2762 questionnaires were finally included in the study set and used in the subsequent statistical analysis.

The questionnaire was composed of categorical, ordinal, and quantitative variables. As a first data inspection, descriptive statistics and nonparametric significance tests [23] were performed according to the variable type. Sex and age were used as stratification variables. According to this, the subjects’ age (in years) was divided into the following four classes: ≤30 years, 31–50 years, 51–64 years, and ≥65 years. A cross-table regarding the bivariate distribution of sex by age in classes was built, and the Chi-square Monte Carlo (MC) test was performed to verify their independence in distribution. The Z-test was then applied to the adjusted Pearson residuals to detect significant (negative or positive) associations between sex and specific age classes. The same procedure, i.e., the construction of a cross-table and a study of the overall distributional independence and “category-by-category” associations through statistical tests, was applied to examine the relationship between, respectively, sex and age in classes with the considered categorical variables (i.e., education degree, job category, smoking habits, and the need for help in improving their lifestyle). Regarding ordinal and quantitative variables (i.e., anthropometric data, hours/night of sleep, total weekly physical activity volume, AHA diet score, perceived somatic symptom scales, and perceived quality scales), descriptive statistics were computed as the median ± MAD (Median Absolute Deviation) within, respectively, sex and age classes and over all the 2762 respondents. The two-tailed Kruskal–Wallis and median MC tests were then performed to verify the absence of differences in these variable distributions between females and males and across age classes.

After these preliminary inspections, statistical analyses focused on the three main study objectives described below.

#### 2.2.1. Subjects’ Intentions Toward Lifestyle Changes

The first objective was to study subjects’ responses to the following question: “In which lifestyle would you most like to improve?”, because it directly expresses subjects’ intentions toward lifestyle changes according to the following single-choice items: eating better, being more physically active, managing stress, improving sleep quality, and stopping smoking.

Assuming that sex and age could have an important role in subjects’ choices, the following analyses were then performed:

- Considering the (marginal) distribution of the response items, the Chi-square test for equal proportions and Z-tests for pairwise comparisons (with Bonferroni *p*-value adjustments) were performed to detect the most prevalent response selected by the respondents;
- Regarding the joint distribution of the response items with sex, the Chi-square MC test for distributional independence was performed along with Z-tests to discover significant associations between sex and subjects’ intentions toward lifestyle changes;
- The same procedure in point b was applied to the joint distribution of the response items with age classes to discover significant associations between age classes and subjects’ intentions toward lifestyle changes;
- Finally, considering the conditional distribution of the response items with sex within age classes, the Chi-square MC test for independence of the response items and sex conditioned on age classes was performed along with Z-tests to discover significant associations between sex and subjects’ intentions toward lifestyle changes for each age class.

#### 2.2.2. Target Actions to Improve Lifestyles and Willingness to Invest Economically in Them

The second objective was to disclose potential associations between the subjects’ real purpose of changing their lifestyle by investing economically in one or more target actions and their actual availability to invest economically in one or more such actions. The target actions were the nine multiple-choice items of the question, “In which of the following activities would you be willing to invest economically to improve your lifestyle?”: buying healthier food, having a medical specialist consultant, buying a gym/swimming pool membership, buying sports equipment/clothing, participating in stress management training, performing medical tests (check-ups), participating in stop-smoking programs, other actions, and no action. Subjects could express their preference for one or more of these multiple-choice items without limitations. On the other hand, the subjects’ availability to invest economically in them was given by the question, “In your opinion, what can be your realistic economic investment in actions to improve your lifestyle?”, with one possible choice among these amount ranges: <EUR 100, EUR 100–299.99, EUR 300–499.99, and ≥EUR 500.

From a statistical point of view, given the multiple-choice structure of the nine target actions to improve lifestyles, each of them was first re-expressed as a binary variable in the form “action *k* chosen: yes/no”, with *k* = 1, …, 9. Then, to detect if there were, in particular, two actions that subjects tended to choose together (excluding the answer “no action”), the strength of association of each pair of these actions was assessed by computing the Phi coefficient (along with 95% confidence intervals) [23]. By referring to conventional rules of thumb, we regarded Phi coefficients that were lower or equal to 0.2 in absolute value as negligible or very weak; in such a case, the two corresponding actions were understood as not being sufficiently strongly associated and were then treated separately in the subsequent analyses.

Besides this, to study the relationship between the nine target actions and the willingness to invest economically in them, for each binary action *k*, a contingency table was built by combining the yes/no responses on action *k* with the amounts subjects intended to invest, thus obtaining nine cross-tables in all. Then, the Chi-square MC test for independence and the Z-tests for the null “action-by-amount” associations were performed on each table.

#### 2.2.3. Profiling of Subjects’ Typologies Through Classification Trees

The third objective was to detect respondents’ profiles that were more likely associated with the following:

- Manifesting a specific intention toward lifestyle changes;
- Choosing at least one target action to improve lifestyles rather than indicating no actions;
- Selecting the two most popular target actions resulted from the survey.

This was a crucial step of the statistical analyses, i.e., identifying the key socio-demographic and behavioral variables and the principal associations among their categories that were capable of profiling specific subject typologies with higher probabilities of having particular desires toward their lifestyle changes (case a), having, or not, actual intentions to make at least one target action to change their lifestyle (case b), and having, or not, particular propensities toward realizing one of the two most popular target actions resulted from the survey (case c).

We relied on classification trees (CTs) based on the recursive partitioning method [19,20] to profile the above subjects’ typologies using all the socio-demographic and behavioral variables involved in the study as predictors, i.e., sex, age in classes, education degree, job category, smoking habits, the need for help for improving their lifestyle, anthropometric and lifestyle variables, perceived somatic symptom scales, and perceived quality scales. To give more substantial meaning to the CTs and, at the same time, simplify the structure of the detected relationships among the variables, we first grouped the values/ordinal scores of the quantitative/ordinal predictors: waist circumference, BMI, lifestyle variables, perceived somatic symptom scales, and perceived quality scales, respectively, into opportunely defined categories and then used such categorized variables as predictors in the CTs. Moreover, the variable “subjects’ intentions toward lifestyle changes”, which is the response variable in the CT of case a, was used as a further predictor in the CTs regarding cases b and c.

We applied the following steps to construct the CTs (all the methodological details are reported in Appendix A):

1. *Pre-processing:* before building the CTs, the goodness of the predictors was assessed in order to discard from the CT construction variables with too low heterogeneity on the subjects (in the extreme case, there would be one single category common to all the subjects) or too high heterogeneity on the subjects (in the extreme case, there would be one single distinct category for each subject);
2. *Pruning:* During the preliminary stage, the CTs were built as wide as possible on the full dataset, with the predictors designated by pre-processing to obtain the largest number of combinations among predictor categories that explained the observed subjects’ class memberships. Then, by the procedure explained in Appendix A, we reduced the CT size and found the best-pruned subtree for each CT to obtain the most important predictors along with their category combinations;
3. *CT validation:* We appraised the performance of the best-pruned subtrees (from here on, more simply indicated as CTs) and the validity of the CT predictors selected by pruning (step 2) from two different perspectives: the CT description quality, which was assessed on the full dataset by also comparing it with the results of a random classifier through a permutation test, and the prediction capability of the selected CT predictors, which was assessed through a bagging procedure implemented by randomly generated training and test sets (see Appendix A) [20]. Both evaluations were carried out by computing the following validation measures based on comparing the subjects’ observed class memberships (i.e., a priori classification) with the predicted ones (i.e., predicted classification) [19]: accuracy (Acc), which gives the total percentage of subjects correctly classified by the CT; sensitivity (Sens), or also the true positive rate, which gives the percentage of subjects with the “positive” event that the CT correctly detects; specificity (Spec), or also the true negative rate, which gives the percentage of subjects with the “negative” event (i.e., the complementary of the “positive” event) that the CT correctly detects; the positive predictive value (PPV), or also precision, which gives the proportion of subjects indicated by the CT as having the positive event that are actually a true positive; and the negative predictive value (NPV), which gives the proportion of subjects indicated by the CT as having negative events that are actually true negatives. We also considered the following two additional measures that are useful for imbalanced data, because they limit the optimistic evaluation of CTs based on accuracy and, at the same time, give importance both to the true positive and true negative rate: balanced accuracy (BalAcc), which is the arithmetic mean of sensitivity and specificity, and the adjusted F-measure (AGF), which is a modification of the F-measures (among which there is F1) introduced to overcome some of their limitations [24].

Throughout the study, the nominal test significance level was set at 0.05. We performed the statistical analysis with the R software, version 4.4.2 [25], along with the following contributed packages: “coin” for the MC version of Kruskal–Wallis and median tests [26]; “ggplot2” [27] for the construction of bar plots; “rpart” [28] for the construction of CTs based on the recursive partitioning method and their pruning; “rpart.plot” [29] for CT graphical representations; “caret” [30] for pre-processing indices, the random generation of training and test sets, and the computation of both variable importance measures of CT predictors and validation measures; “metrica” [31] for the computation of the AGF measure; and “adabag” [32] for the bagging procedure. We followed the STROBE checklist for reporting observational studies to ensure adherence to research standards (see Supplementary Material).

## 3. Results

In the study set composed of 2762 participants, 1107 are females (40.08%), and 1655 are males (59.92%). A total of 83.45% of the respondents are Assidim-associated employees. The overall mean age is 55.09 ± 13.80 years, ranging 20–91 years, with a mean age of 50.68 ± 12.67 years for females (ranging 22–91 years) and 58.03 ± 13.74 years for males (ranging 20–91 years). The distribution of the 2762 participants by sex and age classes is reported in Appendix B Table A1 and Table A2. Most subjects are over 51 years old (64.48%), and, in particular, 25.05% are over 65. However, females and males are distributed significantly differently over the age classes. There are more younger females (females under 51 are 47.33% out of 1107 females) than males (males under 51 are 27.61% out of 1655 males), while there are more males over 65 (33.17% out of 1655) than females (12.92% out of 1107). The entire description of the findings relative to the preliminary data inspection can be found in Appendix B. In particular, Appendix B Table A1 and Table A2 report cross-tabulations and nonparametric test results regarding the categorical variables studied across the four age classes and between females and males, respectively. Appendix B Table A3 and Table A4 include summary statistics and nonparametric test results for the ordinal and quantitative variables studied across the four age classes and between females and males, respectively.

### 3.1. Subjects’ Intentions Toward Lifestyle Changes

**Figure 2:**
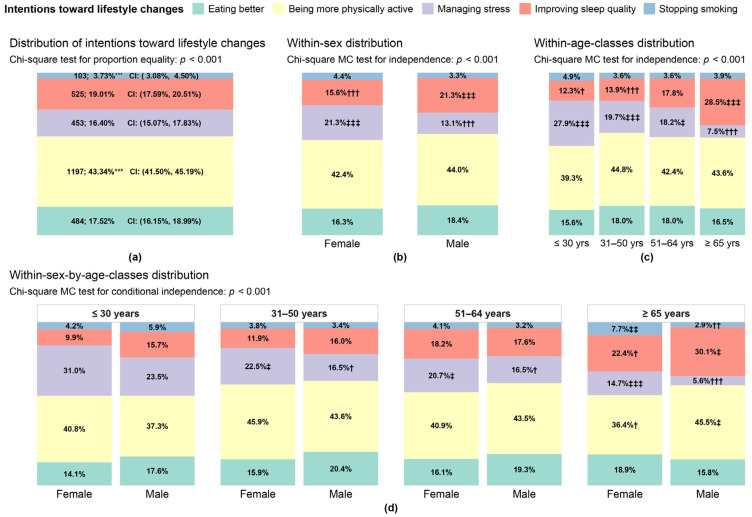
Panels of bar plots of the subjects’ intentions toward lifestyle changes: (**a**) marginal distribution of the five intentions with absolute counts, percentages (computed out of N=2762 respondents), and their 95% confidence intervals (CIs). Significance level codes of the Z-test (with Bonferroni correction) in the pairwise comparisons: $H_{0}:p_{i}\leq p_{r}$ against $H_{1}:p_{i}>p_{r}$, with i≠r=1,…, 5: *** *p* < 0.001. In the pairwise comparisons: $H_{0}:p_{i}\geq p_{r}$ against $H_{1}:p_{i}<p_{r}$, with i≠r=1,…, 5: ^°°°^ *p* < 0.001. (**b**) Within-sex distribution of the five intentions, with conditional percentages computed within females and males, respectively. (**c**) Within-age-classes distribution of the five intentions, with conditional percentages computed within each age class. (**d**) Within-sex-by-age-classes distribution of the five intentions, with conditional percentages computed within sex for each age class. Significance level codes in panels (**b**–**d**) of the Z-test for the single “intention-by-sex” or “intention-by-age-class” association: ^‡^ *p* < 0.05, ^‡‡^ *p* < 0.01, and ^‡‡‡^ *p* < 0.001 (positive association); ^†^ *p* < 0.05, ^††^ *p* < 0.01, and ^†††^ *p* < 0.001 (negative association).

Figure 2 summarizes the results of the analyses concerning the intentions toward lifestyle changes expressed by the respondents. Panel (a) reports the marginal distribution of these responses in terms of absolute counts and percentages, along with 95% confidence intervals. A total of 43.34% (95% CI: (41.50%, 45.19%)) of the subjects declared a desire to become more physically active, 19.01% (95% CI: (17.59%, 20.51%)) to improve sleep hygiene, 17.52% (95% CI: (16.15%, 18.99%)) to have healthier nutrition, 16.40% (95% CI: (15.07%, 17.38%)) to better manage stress, and 3.73% (95% CI: (3.08%, 4.50%)) to stop smoking (which corresponds to 29.01%, considering only the smoking group). The Chi-square test rejected the null hypothesis of equality of these percentages (*p* < 0.001). The comparisons carried out through Z-tests and the Bonferroni correction between each pair of percentages indicate that “becoming physically more active” is the significantly most prevalent response the subjects gave (*p* < 0.001), while, as expected, the significantly least prevalent was “stopping smoking” (*p* < 0.001), since it is only of interest for the smoking group, which is a clear minority in the study set. On the other hand, there are no significant differences in the percentages of respondents concerning the other intentions.

Panel (b) depicts the distribution of the intentions toward lifestyle changes by sex. By the Chi-square test, overall, there are significant differences between females and males regarding the intentions they expressed (*p* < 0.001); such differences regard the choices concerning “managing stress” and “improving sleep quality”, as detected by the Z-tests, while in the other cases, there are no significant “intention-by-sex” associations. Specifically, “managing stress” has a significantly higher percentage in females than in males (21.30% of females vs. 13.10% of males, *p* < 0.001), while “improving sleep quality” has a significantly higher percentage in males than in females (15.60% of females vs. 21.30% of males, *p* < 0.001). Similar to panel (b), panel (c) reports the distribution of the intentions toward lifestyle changes by age classes. In this case, the Chi-square test was also significant (*p* < 0.001), thus indicating that the expressed intentions differ overall across age classes. Once again, the Z-test analysis highlighted that the significant differences concern “managing stress” and “improving sleep quality”, while in the other cases, no significant “intention-by-age” associations were detected. Briefly, “managing stress” has significantly higher percentages in subjects under 65 (27.9% in ≤ 30, 19.7% in 31–50, and 18.2% in 51–64-year-old classes) and a significantly lower percentage in those over 65 (7.5%) than the marginal percentage of 16.40% (panel (a)). Conversely, “improving sleep quality” has a significantly higher percentage in subjects over 65 (28.5%) and significantly lower percentages in those under 51 (12.3% in ≤ 30 and 13.9% in 31–50-year-old classes) than the marginal percentage of 19.01% (panel (a)).

Finally, panel (d) shows the distribution of the expressed intentions by sex, conditioned on age classes. The Chi-square test rejected the overall null hypothesis of independence between intentions and sex, considering age classes (*p* < 0.001). A more in-depth analysis carried out through Z-tests for association showed no significant difference between females’ and males’ intentions in the under-31 class. Conversely, in the 31–50-year-old and 51–64-year-old classes, whilst females and males do not significantly differ concerning eating better, being more physically active, improving sleep quality, and stopping smoking, the percentages of females indicating “managing stress” are significantly higher than males (22.5% of females vs. 16.5% of males in the 31–50-year-old class and 20.7% of females vs. 16.5% of males in the 51–64-year-old class). The majority of significant differences between females and males were detected among those over 65. Females over 65 are characterized by significantly higher percentages of “managing stress” (14.7% vs. 5.6% of males) and “stopping smoking” (7.7% vs. 2.9% of males) and by significantly lower percentages of “being more physically active” (36.4% vs. 45.5% of males) and “improving sleep quality” (22.4% vs. 30.1% of males) than males over 65.

### 3.2. Target Actions to Improve Lifestyles and Subjects’ Willingness to Invest Economically in Them

Table 1 contains the collection of cross-tables of the target actions to improve lifestyles in which subjects intend to invest economically against the actual subjects’ willingness to invest in them according to the given amount ranges. It also reports on the marginal distribution of the economic willingness to invest (last table row) and the number and percentage of subjects choosing and not choosing each specific target action (last two table columns).

Considering the specific actions they are willing to invest in (listed in the order from the most to the least chosen), 35.95% of the subjects indicated having a medical specialist consultant for prescribing a tailored lifestyle program; 35.41% buying a gym/swimming pool membership; 20.82% buying healthier food; 20.09% performing medical tests (check-ups); 14.55% buying sports equipment and clothing; 14.41% participating in stress management training; 8.44% other actions; and 2.79% participating in stop-smoking programs (which corresponds to 21.69% of the smokers). Notably, 298 subjects (10.79% out of 2762) declared that they do not want to invest in any actions to improve their lifestyle, so most (2464 subjects, 89.21%) stated that they are willing to invest in at least one action, among whom, (counts not displayed in Table 1) 1365 subjects indicated only an action (49.42% out of 2762), 621 two actions together (22.48%), 308 three actions together (11.15%), and 170 four actions together (6.15%). The subjects, therefore, chose not more than four actions together.

Two remarks are worth making concerning the multiple-choice structure of the target actions. First, apart from “other actions”, which was a single choice among 70.82% of the subjects indicating it, concerning the two most popular target actions, “buying a gym/swimming pool membership” was a single choice for 40.39% of the subjects choosing it, and “having a medical specialist consultant” was a single choice for 35.45% of the subjects choosing it. This means that both of them are the most selected actions among the subjects and the most frequently occurring as exclusive choices. Second (information not displayed in Table 1), the two most frequently jointly indicated actions were “having a medical specialist consultant” and “buying a gym/swimming pool membership” (95 subjects, 3.44% out of 2762), followed by “having a medical specialist consultant” along with “performing medical tests” (75 subjects, 2.72%). Moreover, the three most frequently jointly indicated actions were “having a medical specialist consultant”, “buying a gym/swimming pool membership”, and “performing medical tests” (37 subjects, 1.34%), while the four most frequently jointly indicated actions were “having a medical specialist consultant”, “buying a gym/swimming pool membership”, “buying healthier food”, and “buying sports equipment and clothing” (54 subjects, 1.96%). Therefore, “having a medical specialist consultant” and “buying a gym/swimming pool membership” were the two most single-choice actions (except for “other actions”) and also the most selected actions, both alone and with the other and in conjunction with other actions.

Nonetheless, although the above-reported percentages of respondents refer to the most frequent multiple choices, they are relatively low; thus, there is high heterogeneity in how the respondents combined their choices of more actions together, which is symptomatic of negligible or weak associations between them. On this point, the Phi coefficient computed for all distinct pairs of target actions (on the subset of 2464 subjects indicating at least one action) gave values that were almost all close to zero or around 0.1 (results not displayed). The only exceptions with Phi coefficients near 0.2 in absolute values regard the negative associations between, respectively, “having a medical specialist consultant” and “other actions” (Phi = −0.195, 95% CI: (−0.233, −0.157)) and “buying a gym/swimming pool membership” and “other actions” (Phi = −0.200, 95% CI: (−0.237, −0.162)). This means that subjects who chose “having a medical specialist consultant” or “buying a gym/swimming pool membership” did not tend to choose “other actions” as well. Since this inspection resulted in no empirical evidence of important associations among target actions, the latter will be treated separately in the subsequent analyses.

Regarding subjects’ economic willingness to invest in actions that are helpful to improve their lifestyle, from Table 1, the last row, 26.03% of the subjects attested their willingness to invest less than EUR 100, 41.17% (i.e., the great majority) from EUR 100 to less than EUR 300, 19.40% from EUR 300 to less than EUR 500, and 13.40% EUR 500 or more. The Chi-square test rejected the independence between economic willingness and target actions in all cases but participating in stress management training and stop-smoking programs. In particular, it is worth noting the significant positive associations between investing less than EUR 100 and indicating no action (30.18% out of 719 vs. 10.79% out of 2762) or other actions (11.27% out of 719 vs. 10.79% out of 2762); investing from EUR 100 to less than EUR 500 and having a medical specialist consultant (41.41% out of 1137 in the range EUR 100–299.99 and 41.98% out of 536 in the range EUR 300–499.99 vs. 35.95% out of 2762); and investing EUR 300 or more and buying a gym/swimming pool membership (42.54% out of 536 within EUR 300–499.99 and 45.14% out of 370 given ≥ EUR 500 vs. 35.41% out of 2762) and/or sports equipment/clothing (19.59% out of 536 within EUR 300–499.99 and 20.81% out of 370 given ≥ EUR 500 vs. 14.55% out of 2762). Therefore, subjects’ economic willingness relates significantly to specific action choices.

### 3.3. Subjects’ Typologies Profiled Through Classification Trees

To best analyze the complex relationships among intentions toward lifestyle changes and willingness to invest economically and in which target actions, considering subjects’ characteristics and actual behavior, in order to also detect specific subject typologies expressing more likely association profiles among the potential predictors, we built CTs to meet the objectives described in Section 2.2.3 by using as response variables, respectively, in case a, the manifested intentions toward lifestyle changes; in case b, the choice of at least one target action against no action; and in case c, the two most selected target actions concerning the investment in a medical specialist consultant, on the one hand, and a gym/swimming pool membership, on the other hand. In particular, since the association between these two actions was negligible, they were separately used as response variables of two distinct CTs.

To build the CTs, we first categorized the values/ordinal scores of the quantitative/ordinal predictors as reported in Appendix B Table A5. The pre-processing assessment evidenced that all the predictors were good enough to be used in the four CTs (Appendix B Table A6, “Pre-processing”). The results described below concern the CTs already pruned and validated, i.e., in their final version.

#### 3.3.1. Classification Tree for Subjects’ Intentions Toward Lifestyle Changes

**Figure 3:**
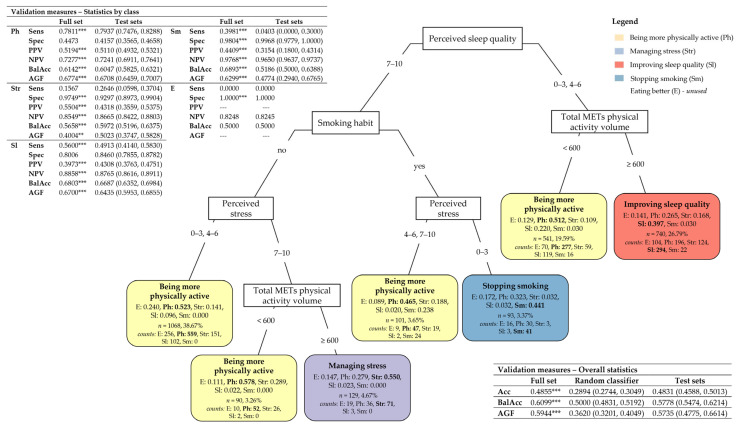
Classification tree of subjects’ intentions toward lifestyle changes. Each leaf node (i.e., terminal CT node) reports the following, in this order: the predicted class (in bold); the probability of each class conditioned on the CT rule (so that the sum of probabilities in each leaf node is 1; the probability in bold pertains to the predicted class and can be regarded as the precision of the prediction in that leaf node); the total count (*n*) and the percentage of subjects that are classified in that leaf node; and the class counts (with the count of the predicted class in bold). The validation measures (overall statistics and statistics by class) relative to the random classifier and the test sets are reported in the two tables inside the graph as means computed over, respectively, the 10,000 permutations of the randomly generated class labels and the 100 repetitions of the bagging procedure, along with 95% confidence intervals built with the percentile method. Significance level codes of the permutation test for comparing the CT built on the full set with the random classifier: ** *p* < 0.01 and *** *p* < 0.001 (Appendix A).

Figure 3 displays the CT for subjects’ intentions toward improving their lifestyle. On pruning, the most important predictors turned out to be the perceived quality of sleep (the CT root node), total MET volume of physical activity, perceived stress, and smoking habits (Appendix B Table A6, “After pruning”). Overall, the CT has a moderately accurate description capability on the full set, 48.55% of the subjects were correctly classified with respect to their intentions (mean on test sets: 48.31%, 95% CI: (45.88%, 50.13%), which denotes a moderate prediction capability of the predictors). Nonetheless, the CT has an overall balanced accuracy significantly greater than 0.5 (mean on test sets: 0.5778, 95% CI: (0.5474, 0.6214)), with a relatively good overall value of the AGF measure, which is around 0.6 (mean on test sets: 0.5735, 95% CI: (0.4775, 0.6614)), and significantly outperforms the random classifier on accuracy, balanced accuracy, and AGF (*p* < 0.001). Moreover, the CT is highly sensitive to the intention to be more physically active: 78.11% of subjects manifesting this desire were correctly classified (mean on test sets: 79.37%, 95% CI: (74.76%, 82.88%); BalAcc = 0.6047, 95% CI: (0.5825, 0.6321); sensitivity of the random classifier (not displayed in Figure 3) = 45.60%, 95% CI: (43.53%, 47.70%), *p* < 0.001), with a precision equal to 51.94% (mean on test sets: 51.10%, 95% CI: (49.32%, 53.21%)). To a lesser extent, the CT also has relatively good sensitivity for “improving sleep quality”, with a true positive rate of 56% (mean on test sets: 49.13%, 95% CI: (41.40%, 58.30%); BalAcc = 0.6687, 95% CI: (0.6352, 0.6984); sensitivity of the random classifier (not displayed in Figure 3) = 19.50%, 95% CI: (16.57%, 22.48%), *p* < 0.001), with a precision equal to 39.73% (mean on test sets: 43.08%, 95% CI: (37.63%, 47.51%)]. On the other hand, the CT has poor sensitivity for “managing stress” (only 15.67% of the subjects manifesting this desire were truly detected; no significant difference occurs with the random classifier) and null sensitivity for “eating better”, thus indicating that the available predictors are at all not capable of intercepting this intention among subjects.

Based on the CT partitioning rules depicted in Figure 3, it is possible to make the following comments regarding the resulting subjects’ profiling:

- Among the subjects declaring to perceive a high quality of sleep (7–10, left branch), those who are non-smokers and with a low–medium level of perceived stress (0–3, 4–6) have a higher probability (estimated at 0.523) of desiring to be more physically active than the other intentions. The subjects also declaring a high level of perceived stress (7–10) and being a little physically active (total METs < 600) have a higher probability (estimated at 0.578) of desiring to be more physically active. However, if they are already physically active (total METs ≥ 600), they are more likely to prefer to focus on managing stress (with a probability estimated at 0.550). Perceived stress plays an important role in the case of smokers as well. If these subjects perceive a medium–high stress level (4–6, 7–10), they are more likely to desire to be more physically active (with a probability estimated at 0.465), while if their perceived level of stress is low (0–3), they are more likely to intend to quit smoking (with a probability estimated at 0.441);
- Among the subjects declaring to perceive a low–medium quality of sleep (0–3, 4–6, right branch), the total MET physical activity volume results in the unique most important predictor, subjects who are little physically active (total METs < 600), are more likely to desire to be more physically active (with a probability estimated at 0.512). In contrast, those already physically active (total METs ≥ 600) have a higher probability (estimated at 0.397) of desiring to improve sleep quality.

#### 3.3.2. Classification Tree of “At Least One Target Action” Against “No Action”

**Figure 4:**
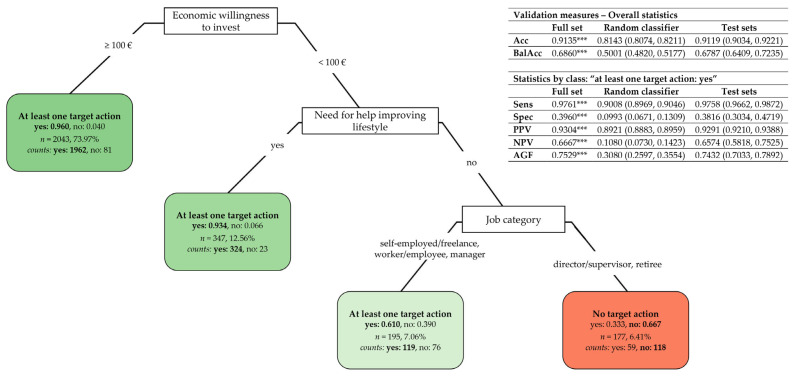
Classification tree of at least one target action against no action in which subjects intend to invest economically. The meaning of the quantities appearing in the graph is explained below in Figure 3. Significance level code of the permutation test for comparing the CT built on the full set with the random classifier: *** *p* < 0.001 (Appendix A).

Figure 4 shows the CT for subjects choosing at least one target action in which they intend to invest economically against choosing no action. The most important predictors resulted in the economic willingness to invest (the CT root node), the need for help in improving their lifestyle, and job category (Appendix B Table A6, “After pruning”). Overall, the CT has a strongly accurate description capability, since 91.35% of the subjects were correctly classified on the full set (mean on test sets: 91.19%, 95% CI: (90.34%, 92.21%), which indicates a strong prediction capability of the predictors), with an overall balanced accuracy significantly greater than 0.5 (mean on test sets: 0.6787, 95% CI: (0.6409, 0.7235)). Moreover, the CT is highly sensitive for “at least one action”, with a true positive rate of 97.61% (mean on test sets: 97.58%, 95% CI: (96.62%, 98.72%)) and a precision of 93.04% (mean on test sets: 92.91%, 95% CI: (92.10%, 93.88%)). It has, however, low specificity: only 39.60% of subjects declaring no action were truly detected (mean on test sets: 38.16%, 95% CI: (30.34%, 47.19%)) with a negative predictive value equal to 66.67% (mean on test sets: 65.74%, 95% CI: (58.18%, 75.25%)). Nonetheless, the CT has a good value of the AGF measure (mean on test sets: 0.7432, 95% CI: (0.7033, 0.7892)) and outperformed the random classifier significantly on all the considered validation measures (*p* < 0.001).

The CT partitioning rules in Figure 4 evidence the following subjects’ typologies:

- Subjects willing to invest EUR 100 or more (left branch) have a very high probability (estimated at 0.960) of choosing at least one target action;
- Among the subjects willing to invest less than EUR 100 (right branch), those declaring they need help in improving their lifestyle have a higher probability (estimated at 0.934) of choosing at least one action. Otherwise, job category becomes an important predictor: directors/supervisors and retirees are more likely to choose no action (with a probability estimated at 0.667), while other job categories are associated with a higher probability (estimated at 0.610) of selecting at least one action.

#### 3.3.3. Classification Tree of Target Action “Having a Medical Specialist Consultancy”

**Figure 5:**
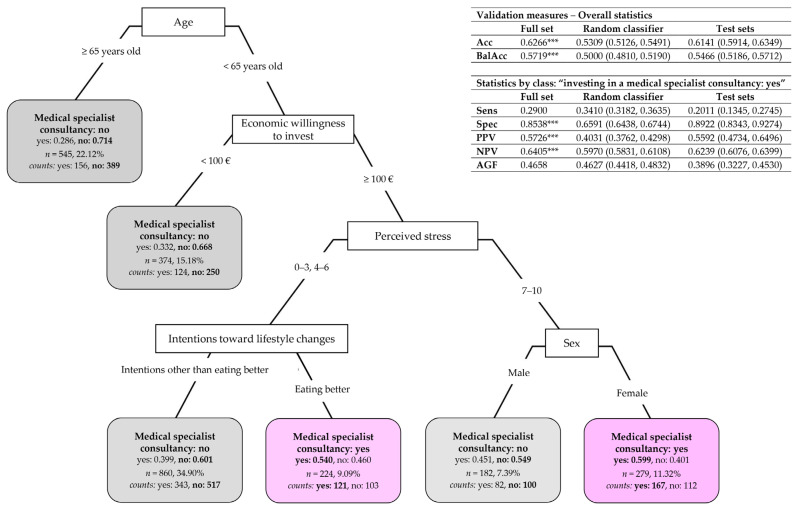
Classification tree of the target action “investing in a medical specialist consultancy for a tailored lifestyle program” chosen by subjects among the 2464 indicating at least one action in which to invest economically. The meaning of the quantities appearing in the graph is explained below in Figure 3. Significance level code of the permutation test for comparing the CT built on the full set with the random classifier: *** *p* < 0.001 (Appendix A).

Figure 5 depicts the CT for subjects choosing to invest in a medical specialist consultant for a tailored lifestyle program. This CT was built over the subset of 2464 subjects who indicated at least one target action in which to invest economically. Five main predictors resulted from pruning: age (the CT root node), the economic willingness to invest, perceived stress, subjects’ intentions toward lifestyle changes, and sex (Appendix B Table A6, “After pruning”). Overall, the CT has a medium–high accuracy in its description capability: a total of 62.66% of the subjects were correctly classified on the full set according to whether they intend or not to have a medical specialist consultant (mean on test sets: 61.41%, 95% CI: (59.14%, 63.49%), which indicates a medium–high prediction capability of the predictors), with an overall balanced accuracy significantly greater than 0.5 (mean on test sets: 0.5466, 95% CI: (0.5186, 0.5712)). Nonetheless, the CT has a high specificity toward “not having a medical specialist consultant” (85.38% of subjects truly recognized with a negative predictive value of 64.05%; mean on test sets: 89.22%, 95% CI: (83.43%, 92.74%), for specificity, and 62.39%, 95% CI: (60.76%, 63.99%), for NPV) and a low sensitivity toward “having a medical specialist consultant” (29% of subjects truly detected with a precision of 57.26%; mean on test sets: 20.11%, 95% CI: (13.45%, 27.45%), for sensitivity, and 55.92%, 95% CI: (47.34%, 64.96%), for PPV), with a relatively low value of the AGF measure (mean on test sets: 0.3896, 95% CI: (0.3227, 0.4530)). The CT significantly outperformed the random classifier on accuracy, balanced accuracy, specificity, PPV, and NPV (*p* < 0.001) but not on sensitivity and AGF.

The CT partitioning rules in Figure 5 yield the following subjects’ typologies:

- Subjects over 65 (left branch) have a very high probability (estimated at 0.714) of not choosing to have a medical specialist consultant;
- Among the subjects under 65 (right branch), those declaring they are willing to invest less than EUR 100 are more likely to not be interested in having a medical specialist consultant (with a probability estimated at 0.668). Otherwise, perceived stress plays an important role in subjects willing to invest EUR 100 or more. In particular, on the one hand, if subjects have a low–medium level of perceived stress (0–3, 4–6) and intend to eat better, then they are more likely to invest in a medical specialist consultant (with a probability estimated at 0.540); otherwise, if they have intentions other than eating better, they are more likely to be uninterested in having a medical specialist consultant (with a probability estimated at 0.601). On the other hand, if subjects have a high level of perceived stress (7–10) and are females, they are more likely to invest in a medical specialist consultant (with a probability estimated at 0.599). In contrast, males have a higher probability (estimated at 0.549) of not investing in a medical specialist consultant.

#### 3.3.4. Classification Tree of Target Action “Buying a Gym/Swimming Pool Membership”

**Figure 6:**
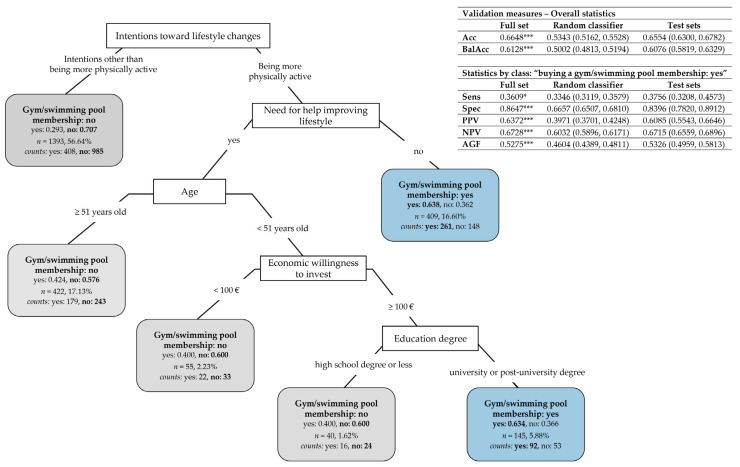
Classification tree of the target action “buying a gym/swimming pool membership” chosen by subjects among the 2464 indicating at least one action in which to invest economically. The meaning of the quantities appearing in the graph is explained below in Figure 3. Significance level codes of the permutation test for comparing the CT built on the full set with the random classifier: * *p* < 0.05 and *** *p* < 0.001 (Appendix A).

Figure 6 reports the CT for subjects choosing to invest in a gym/swimming pool membership. Again, this CT was built over the subset of 2464 subjects who indicated at least one target action in which to invest economically. Once again, five main predictors resulted from pruning: subjects’ intentions toward lifestyle changes (the CT root node), the need for help in improving their lifestyle, age, the economic willingness to invest, and education degree (Appendix B Table A6, “After pruning”).

Overall, the CT has a medium–high accuracy in its description capability: a total of 66.48% of the subjects were correctly classified on the full set according to whether they intend or not to buy a gym/swimming pool membership (mean on test sets: 65.54%, 95% CI: (63.00%, 67.82%), which denotes a medium–high prediction capability of the predictors), with an overall balanced accuracy significantly greater than 0.5 (mean on test sets: 0.6076, 95% CI: (0.5819, 0.6329)). However, the CT has a high specificity toward “not buying a gym/swimming pool membership” (86.47% of subjects correctly classified with a negative predictive value of 67.28%; mean on test sets: 83.96%, 95% CI: (78.20%, 89.12%), for specificity, and 67.15%, 95% CI: (65.59%, 68.96%), for NPV) and a low sensitivity toward “buying a gym/swimming pool membership” (36.09% of subjects correctly classified with a precision of 63.72%; mean on test sets: 37.56%, 95% CI: (32.08%, 45.73%), for sensitivity, and 60.85%, 95% CI: (55.43%, 66.46%), for PPV), with a medium value of the AGF measure (mean on test sets: 0.5326, 95% CI: (0.4959, 0.5813)). The CT significantly outperformed the random classifier on accuracy, balanced accuracy, specificity, PPV, NPV, and AGF (*p* < 0.001), as well as on sensitivity (*p* < 0.05).

The CT partitioning rules in Figure 6 lead to the following subjects’ typologies:

- Subjects who have intentions other than becoming more physically active (left branch) have a very high probability (estimated at 0.707) of not investing in a gym/swimming pool membership;
- Among the subjects who intend to be more physically active (right branch), those declaring they need no help in improving their lifestyle are more likely to buy a gym/swimming pool membership (with an estimated probability of 0.638). Otherwise, the other three predictors become important among subjects needing help in improving their lifestyle. In particular, subjects over 51 are more likely not to invest in a gym/swimming pool membership (with an estimated probability of 0.576). On the contrary, in subjects under 51, this investment depends, above all, on their economic willingness, so they are more likely to not buy a gym/swimming pool membership if their willing amount of investment is less than EUR 100 (with a probability estimated at 0.600). In the case of subjects willing to invest more than EUR 100, their choice for buying, or not, a gym/swimming pool membership depends on their education degree: if they are graduates or postgraduates, they are more inclined to invest in a gym/swimming pool membership (with a probability estimated at 0.634); otherwise, they are not (with an estimated probability of 0.600).

## 4. Discussion

The present study proposes new information potentially useful in designing campaigns to foster well-being and prevent chronic diseases, particularly to tailor interventions that help improve lifestyles based on subjects’ characteristics and desires. Using an anonymous, web-based questionnaire, we focused on analyzing subjects’ intentions toward lifestyle changes and their willingness to invest economically in one or more of the tools/actions indicated in the survey. We observed that respondents’ main lifestyle improvement desire is to become more physically active (both in males and females of any considered age) (see Figure 2) and that the most preferred tools chosen to improve their lifestyle are the possibility of having a medical specialist consultant to prescribe a tailored lifestyle program and buying a gym/swimming pool membership.

A more sophisticated statistical approach based on classification trees [19,20] permitted the unveiling of specific subjects’ typologies characterized by a higher probability of desiring to be more physically active or willing to invest in buying a medical specialist consultant or a gym/swimming pool membership. Implicitly, the classification trees allowed for the most important predictors and the associations among their categories to be disclosed, based on which subjects’ typologies were defined. This included the following, in particular:

- The perceived quality of sleep, the total volume of physical activity, smoking habits, and perceived stress resulted in the most important predictors that might explain subjects’ intentions of being more physically active (Figure 3);
- The economic willingness to invest, the need, or not, for help in improving their lifestyle, and job category are the most important selected predictors that might explain subjects’ willingness to invest in at least one target action (Figure 4);
- Age, the economic willingness to invest, perceived stress, the intention to eat better, and sex are the most important predictors that might explain subjects’ willingness to invest in a medical specialist consultant (Figure 5);
- The intention to be more physically active, the need, or not, for help in improving their lifestyle, age, the economic willingness to invest, and education degree are the most important predictors that might explain subjects’ willingness to invest in a gym/swimming pool membership (Figure 6).

Besides this, it is worth summing up the main associations among predictor categories resulting from the study, which correspond to specific subject typologies:

- Subjects characterized by a higher probability of desiring to become more physically active (Figure 3) are those who perceive a low–medium level of sleep quality (0–3, 4–6) and do not meet the WHO’s recommended dose of aerobic exercise (total METs < 600); or have a very positive perception of sleep quality (7–10) but are smokers with a medium–high level of perceived stress (4–6, 7–10); or are non-smokers with a low–medium level of perceived stress (0–3, 4–6); or are non-smokers with high-stress perception (7–10) not meeting the WHO’s recommended dose of aerobic exercise (total METs < 600);
- Subjects characterized by a higher probability of being willing to invest in at least one target action to improve their lifestyle (Figure 4) are those who indicate a willingness to invest more than EUR 100; or among those willing to invest less than EUR 100 are those needing help to improve their lifestyle; or are those needing no help and being employed in specific job categories (i.e., self-employed/freelancers, workers/employees, and managers);
- Subjects characterized by a higher probability of willing to invest in a medical specialist consultant for receiving a tailored prescription of a lifestyle program (Figure 5) are those under 65 and willing to invest more than EUR 100, with a low–medium level of perceived stress (0–3, 4–6) and the desire to have healthier nutrition; or are women under 65 that are willing to invest more than EUR 100 and who have a high level of perceived stress (7–10);
- Subjects characterized by a higher probability of buying a gym/swimming pool membership (Figure 6) are those who desire to become more physically active and declare no need for help in improving their lifestyle; or, among the subjects needing help, those under 51 declaring to be willing to invest more than EUR 100 and who are graduates or postgraduates.

A crucial aspect of this study was to inspect the role of sex and age as explicative of manifesting particular desires toward lifestyle changes or having or not having particular propensities toward specific actions to take to achieve their goals. The preliminary statistical analysis of the socio-demographic and behavioral variables across the sex and age classes evidenced noteworthy differences. Notably, females needing help in improving their lifestyle were in a significantly higher percentage than males, while no significant difference was observed across age classes (see Appendix B Table A1 and Table A2). Moreover, females had higher levels than males on the perceived somatic symptom scales (short 4SQ, fatigue, and stress), and similarly, the youngest subjects had higher levels than the oldest subjects on such scales. Moreover, no significant differences were detected in the perceived quality scales (sleep, health, and job performance) between females and males and across age classes, except for the perceived quality scale of job performance, which had the lowest level in the oldest subjects (see Appendix B Table A3 and Table A4). The results in Figure 2 about subjects’ intentions toward lifestyle changes further evidenced differences between females and males and across age classes (panels (b)–(d)). In particular, females over 31 years old intending to manage stress were in significantly higher percentages than males, while the percentage of males over 65 desiring to improve sleep quality was significantly higher than that of females (panel (d)). However, when sex and age were used as predictors in the presence of other predictors in the classification tree of subjects’ intentions toward lifestyle changes, they resulted as less important than the perceived quality of sleep, the total volume of physical activity, smoking habits, and perceived stress (see Appendix B Table A6). Consequently, they do not appear in the pruned classification tree in Figure 3. In particular, the intention of managing stress is more likely to be present in subjects who are non-smokers, physically active, and have high perceived sleep quality but with high perceived stress. At the same time, the intention of improving sleep quality is more likely to be present in subjects who are physically active and have medium–low perceived sleep quality. Nonetheless, sex and, above all, age resulted as fundamental predictors in the classification tree of the target action “investing in a medical specialist consultant for a tailored lifestyle program” (see Figure 5). Age also appeared to be among the most important predictors in the classification tree of “buying a gym/swimming pool membership” (see Figure 6).

The workplace may play a pivotal role in the prevention of chronic diseases and in fostering well-being [1,7,8,9,10,11,12,13,14]. Strategies employed to this end vary from general educational campaigns on chronic diseases, cardiometabolic risk factors, and healthy lifestyles to offering health check-ups aiming at the early detection of signs of diseases (for instance, breast or colon cancer) or at the definition of traditional cardiometabolic risk factors (such as lipid profiles, plasma glucose levels, arterial pressure levels, etc.) [33,34,35]. The scientific literature and insurance data nevertheless show that attendance to health check-ups is lower than desired [36], particularly in young people [37,38,39,40], and they even fail to prevent chronic diseases when not followed by changes in lifestyles and risks [36,41,42,43]. In this paper, we observed that performing medical tests (check-ups) was only indicated in 20.09% of the cases among the preferred tools considered important to prevent diseases and improve lifestyles (Table 1), corroborating the observation of the reduced adherence to campaigns that promote them. On the other hand, surprisingly, we observed that our study population mainly indicated the willingness to invest in having a medical specialist consultant to receive a tailored prescription for a lifestyle modification program (35.95%) and in buying a gym/swimming pool membership (35.41%), considering these tools more useful to better their lifestyle (Table 1). These data might be taken into consideration by companies and insurance agencies when offering subjects benefits and/or campaigns to foster well-being and prevent chronic diseases, moving some investments from costs devoted to medical assessments (check-ups) to interventions aiming to prescribe tailored programs to improve lifestyles.

Notably, with the obvious exception of subjects unwilling to devote more than EUR 100, there was a desire to receive a medical specialist consultant for a tailored program independently of the other amounts of economic availability. Moreover, this action was chosen particularly by subjects under 65, as indicated in Figure 5 (right branch of the classification tree). This observation might be elucidated by taking into consideration a result of our previous paper showing that older subjects are characterized by a better lifestyle [3], then perceiving less need to be helped in improving it; nevertheless, the classification tree approach also considered predictors linked to individual behaviors (such as the dose of performed exercise, quality of nutrition, smoking, etc.). Notably, age was also a determinant of the willingness to buy a gym/swimming pool membership (Figure 6), close to economic availability and education degree, in the latter case, confirming observations present in the literature on both Asian [44] and European populations [45] that depicted a link between a high educational level and exercise.

The perception of stress merits a comment as a predictor of the desire to be more physically active (Figure 3) and the willingness to invest in medical consultation (Figure 5). Stress is nowadays of main concern worldwide, affecting well-being at any age, in particular in youth [46] and employees [47], and stress management initiatives are welcomed as strategies to improve and maintain health [46,48,49,50,51]. In previous papers, we observed high-stress perception in young employees, particularly in women [3] and less active subjects [6]. In this paper, we confirm these findings on a different, wider population (see Appendix B Table A3 and Table A4) and add the observation that young and female participants declared the desire to manage stress (see Figure 2). Nevertheless, a more advanced statistical approach aiming to unveil characteristics of specific groups of subjects to offer tailored lifestyle intervention programs showed that exercise was chosen independently of age and sex as a possible strategy to improve their lifestyle by participants characterized by high-stress perception (see Figure 3) and that only already fit subjects chosen stress management strategy. These data may greatly impact solutions proposed to improve well-being, particularly by institutions such as large companies or universities, to avoid ineffective programs. To corroborate this issue, we report recent data from our group [2] which unveiled, in a large cohort of undergraduate students who voluntarily participated in a lifestyle survey, three different clusters: one (A) of fit and with low-stress perception students, one (B) of unfit and with high-stress perception students, and one (C) of fit and with high-stress perception students. The two latter clusters might deserve a different approach to managing stress, being the simple promotion of exercise not indicated for cluster C, while stress management courses, educational sections, etc., might be more useful. Stress management may consider different strategies, from more traditional psychological approaches, such as mindfulness and cognitive restructuring of stress to lifestyle modifications [52]. The results of our study might help address specific strategies for specific subject groups, possibly favoring the success of the intervention.

Smoking also needs to be considered. Our results (see Figure 3) show that only smoker respondents who perceived a low level of stress desired to stop smoking, while those characterized by a high-stress perception desired to become more physically active, corroborating the role of exercise as a tool to manage stress.

Nutrition represents another fundamental aspect of fostering well-being and health and preventing/managing many chronic diseases. In this study, we assessed both anthropometric data affected by nutritional patterns (such as BMI and waist circumference) and the quality of nutrition employing the AHA diet score [22] (Appendix B Table A3 and Table A4). We enquired about the willingness to improve nutrition quality (as a desired lifestyle to improve well-being; see Figure 2) and to buy healthier food (as a target action to improve lifestyles; see Table 1). We did not ask directly about the desire to lose weight, because this requires a complex approach, including actions aimed at improving the quality and quantity of food, increasing the exercise dose, and even managing stress. Our results unveil that participants indicated a desire to eat better, independently of sex and age, in a relatively small percentage (around 17 participants out of 100; see Figure 2). The notable classification tree approach showed that the desire to have nutrition of better quality characterized a specific group of respondents: those who are willing to invest in medical consultation to receive a tailored prescription, aged less than 65 years, declaring to be willing to invest more than EUR 100, and presenting low–medium stress perception (see Figure 5). The desire to eat better was also involved in the construction of the other classification trees, respectively, as a category to be predicted in the multi-class problem of Figure 3 and as a category of the predictor “subjects’ intentions toward lifestyle changes” used in Figure 4 (“at least one target action” versus “no target action”) and Figure 6 (“buying a gym/swimming pool membership”). However, the performed analyses evidenced no other meaningful result for the desire to eat better. In particular, the null sensitivity for “eating better” that resulted in the classification tree for the subjects’ intentions toward lifestyle changes (Figure 3) evidences that intercepting this specific intention is more complex than using the socio-demographic and behavioral predictors considered in this study; this would deserve an ad hoc approach which should also take into account subjects’ willingness to lose weight with all the actions necessary to reach this goal.

Another aspect that deserves comment is the importance of sleep [53,54]. Sleep quality is indicated as an important intention for lifestyle improvement, particularly in older male participants (see Figure 2). It moreover represents the principal predictor of the desire to be more physically active (see Figure 3): even subjects who reported a low–medium sleep quality and were unfit declared the desire to become physically active. These findings might be important in strengthening the use of exercise as a strategy to improve sleep, as indicated by several scientific papers [55,56], and this action might be worthwhile in elders. Improving poor sleep quality, considering its impact on well-being and quality of life, might be a motivational tool for this specific age group to become more physically active and simultaneously obtain all the benefits associated with this healthy behavior. Vice versa, the same motivational role might be at play for the reduction in stress perception in young subjects.

Commenting on a result shown in Figure 4 may also be valuable. Job categories resulted in an important predictor of the economic willingness to invest, or not, in at least one target action to improve lifestyles. Among the subjects unwilling to invest more than EUR 100 and declaring no need for help in improving their lifestyle (right branch of the classification tree), lower-level job positions seemed more inclined to invest in at least one action than higher-level job positions (directors/supervisors) and retirees. This finding may be explained by considering that subjects with lower-level positions might be interested in improving their lifestyle despite their low declared economic availability. On the contrary, subjects with higher-level positions or who are retired were more likely to declare themselves uninterested in investing in any action, suggesting that their willingness to invest less than EUR 100 was not linked to economic restrictions but to an apparent disinterest in investing in any action addressed to improve their lifestyle. This result might further corroborate the utility of classification trees in unveiling specific subject groups based on the meaningful associations among categories of socio-demographic and behavioral variables, which can be read straight at the different branches and nodes of the trees.

### Limitations

We have to acknowledge some limitations of our study. The first one is that data were obtained by self-reported questionnaires, which could be considered of suboptimal quality. However, the elevated number of respondents and the preliminary exhaustive data quality analysis may have reduced the impact of this possible limitation. In addition, although the questionnaire was completely anonymous, it nevertheless provided each participant with personalized, immediate feedback based on the provided information. In a previous study, we showed that this approach increased participants’ compliance in inputting reliable data [18] to obtain a report reflecting their condition. Moreover, using the present survey performed in 2024, we confirmed some important results that we already described in previous papers [3,6] for a different population. Secondly, we are fully aware that the proposed target actions are limited and not exhaustive, so much so that we considered the option “other actions” or “no action” to permit every respondent to indicate an option. Table 1 shows that these two options were chosen primarily by the group of respondents with low economic availability and were less represented in other groups. Nevertheless, this remains a limitation that cannot be overcome, because it was not possible to include all the possible target actions. Third, these findings are limited to the studied population and may not be generalized to populations with far different characteristics, because respondents voluntarily filled in the questionnaire, and no probabilistic sampling schema could be adopted. However, the high number of respondents considered in the statistical analyses and the advanced methodology used to validate the classification trees could soften the impact of auto-selected respondents on the results achieved. In addition, such findings are specifically intended to be helpful for selected companies so that they can propose strategies to improve their employees’ lifestyles and foster well-being tailored to their specific characteristics. The classification tree results should be regarded just from this perspective: although the validation measures, especially sensitivity, did not achieve the typical high values reported in biostatistics studies (e.g., where the effectiveness of a new drug or a new diagnostic test is assessed), the classification trees helped, all the same, identify subjects’ typologies in terms of potential associations among categories of socio-demographic and behavioral variables that are more likely to manifest specific desires or intentions concerning the actions to take to improve lifestyles. At the same time, the validity of the subjects’ typologies detected in our study should be regarded with caution. Although the classification trees were built using a rigorous statistical procedure, including the construction of random classifiers as references for performance comparisons and the bagging procedure to assess the prediction capability of the selected predictors, they are strongly tied to the specific population studied. Generalizing these findings to populations with far different characteristics could be misleading. In this light, while some validation measures for the classification trees may show high values, they should not be interpreted as having general or absolute validity. Further research should focus on developing a broader and more robust profiling of subjects’ typologies by including a wider population with a greater range of diverse characteristics. Moreover, we had to consider that due to privacy reasons (in our country), even if the questionnaire was anonymous, it was not possible to render such a lifestyle assessment mandatory for all the subjects. Finally, we could not perform individual clinical assessments, such as personalized cardiopulmonary assessments [57], which may be recommended for further clinical research, considering they may also be affected by lifestyles [58].

## 5. Conclusions

In conclusion, this research might help tailor worksite health promotion interventions and insurance services offered to working people, initiatives that may play an important role in fostering health/well-being and preventing chronic diseases. The practical message of our study is that the declared desire to improve one’s lifestyle needs to match practical actions, not limited to health check-ups but also by considering defining tailored, personalized programs and instrumental actions to realize them. Moreover, important individual/group characteristics need to be taken into account.

## Figures and Tables

**Table 1:** Collection of cross-tables of the target actions to improve lifestyles by the subjects’ economic willingness to invest in them, with significance tests for independence and category-by-category associations ^1^.

	Economic Willingness to Invest (One Choice)					
**Target Actions to Improve Lifestyles (Multiple Choices)**	**<100 €**	**100–299.99 €**	**300–499.99 €**	**≥500 €**	***Total Number of Respondents Choosing the Action***	***Total Number of Respondents Not Choosing the Action***
Buying healthier food ^†††^	78 (*10.85%*) ***	264 (23.22%) **	137 (25.56%) **	96 (25.95%) **	575 (20.82%) s.c.: 17.39%	2187 (79.18%)
Having a medical specialist consultant ^†††^	153 (*21.28%*) ***	471 (41.41%) ***	225 (41.98%) ***	144 (38.92%)	993 (35.95%) s.c.: 35.45%	1769 (64.05%)
Buying a gym/swimming pool membership ^†††^	162 (*22.53%*) ***	421 (37.03%)	228 (42.54%) ***	167 (45.14%) ***	978 (35.41%) s.c.: 40.39%	1784 (64.59%)
Buying sports equipment/clothing ^†††^	61 (*8.48%*) ***	159 (13.98%)	105 (19.59%) ***	77 (20.81%) ***	402 (14.55%) s.c.: 23.88%	2360 (85.45%)
Participating in stress management training	85 (11.82%)	182 (16.01%)	74 (13.81%)	57 (15.41%)	398 (14.41%) s.c.: 30.40%	2364 (85.59%)
Performing medical tests (check-ups) ^†††^	77 (*10.71%*) ***	232 (20.40%)	137 (25.56%) ***	109 (29.46%) ***	555 (20.09%) s.c.: 19.64%	2207 (79.91%)
Participating in stop-smoking programs	19 (2.64%)	37 (3.25%)	13 (2.43%)	8 (2.16%)	77 (2.79%) s.c.: 35.06%	2685 (97.21%)
Other actions ^†^	81 (11.27%) ***	84 (*7.39%*) *	37 (6.90%)	31 (8.38%)	233 (8.44%) s.c.: 70.82%	2529 (91.56%)
No action ^†††^	217 (30.18%) ***	45 (*3.96%*) ***	12 (*2.24%*) ***	24 (*6.49%*) **	298 (10.79%)	2464 (89.21%)
***Distribution of economic willingness to invest*** **(*% out of 2762 subjects*)**	719 (26.03%)	1137 (41.17%)	536 (19.40%)	370 (13.40%)		

^1^ For simplicity, Table 1 shows, in its rows, only the part of the nine contingency tables in which action *k* is associated with the answer “yes”. The “Total number of respondents choosing the action” column reports the number $n_{k}$ of respondents that chose action *k*; this number is the sum of the counts in the *k*-th row. Vice versa, the “Total number of respondents not choosing the action” column reports the complementary counts, $N-n_{k}$, of respondents that did not choose action *k*. The percentages reported in round brackets below these two total counts, i.e., $p_{k}$ and $100-p_{k}$, respectively, were computed out of the N=2762 respondents. Conversely, the $p_{k|j}$ percentages reported in the *k*-th row within the *j*-th column (in round brackets below the counts in the first four columns) were computed out of the marginal counts, $n_{j}$, of the amount ranges of the economic willingness to invest (last table row). This way, the $p_{k|j}$ percentages can be directly compared with their counterpart percentages, $p_{k}$, reported in the “Total number of respondents choosing the action” column, so that the positive/negative associations between pairs of responses can be noted more straightforwardly. Moreover, the third value reported in the cells of the “Total number of respondents choosing the action” column and labeled as “s.c.”, i.e., single-choice, is the percentage of the $n_{k}$ subjects choosing action *k* without choosing any further actions. Significance level codes for the Chi-square MC test for the independence (based on 10,000 samples) of each target action on the economic willingness to invest: ^†^ *p* < 0.05, and ^†††^ *p* < 0.001. Significance level codes for the Z-test for the single “action-by-amount” association (based on the standardized normal distribution of the adjusted Pearson residuals): * *p* < 0.05, ** *p* < 0.01, and *** *p* < 0.001. Percentages denoting significant positive “action-by-amount” associations (i.e., significantly greater than the expected percentages) are in bold. Percentages denoting significant negative “action-by-amount” associations (i.e., significantly lower than the expected percentages) are in italics.

## Data Availability

Data will be uploaded if the paper is accepted for publication. This raw dataset will be accessible upon request, because it includes sensitive information. Requests can be addressed to daniela.lucini@unimi.it.

## Supplementary Materials

The following supporting information can be downloaded at: https://www.mdpi.com/article/10.3390/nu17111819/s1, STROBE Statement—checklist.

## Appendix A

Methodological details concerning the three steps in constructing the CTs:

1. *Pre-processing:* To assess the goodness of the predictors, we computed two indices for each: (A) a “frequency ratio”, i.e., the ratio between the frequency of the most prevalent category over the second most prevalent category. Predictors with such a ratio above the cutoff of 95/5 were discarded from the CT construction, because they were considered to be of too low heterogeneity on the subjects; (B) a “percentage of unique categories”, i.e., the percentage of distinct categories out of the total number (*N* = 2762) of subjects. Predictors with such a percentage above the cutoff of 10 were discarded from the CT construction, because they were considered to be of too high heterogeneity in the subjects.
2. *Pruning:* During the preliminary stage, the CTs were built as wide as possible on the full dataset, with the so-called complexity parameter set to 0 [19] to obtain the largest number of combinations among predictor categories that explained the observed subjects’ class memberships. In order to avoid, however, building CTs with too huge a dimensionality, the minimum number of subjects that had to exist in a CT node for a new split to be conducted was set to 30, and the minimum number of subjects in each CT leaf (i.e., a terminal CT node) was set to 10. Then, we assessed the best value for the complexity parameter through a 10-fold cross-validation procedure along with the “1 standard error rule” [19,20] to find the best-pruned subtree for each CT.
3. *CT validation:* We carried out a bagging procedure [20] to assess the performance of the best-pruned subtrees derived from the above procedure (from here on, more simply indicated as CTs) from the perspective of the prediction capability of the selected CT predictors. To apply bagging, which stands for “bootstrap aggregation”, we first randomly split the full dataset into a training set with 70% of the subjects and a test set with 30% of the subjects. We applied a stratified random sampling scheme to ensure that all the categories of the CT response variables were present in the training and test sets (approximately in the same proportions as the full dataset). Second, by the bagging approach, we generated *B* = 200 bootstrap samples of the training set, and a given CT was trained on them to obtain 200 bagged CTs. Then, for a given subject (in the training set or test set), 200 different predictions of his/her class membership on the CT response variable were available so that, by the principle of the majority vote [20], his/her unique predicted class membership was given as the most commonly predicted class among the 200 predictions. This bagging procedure was repeated 100 times from the random generation of training and test sets to predicting each subject’s class membership. To evaluate the CT performance in terms of both description and prediction capabilities, we computed the following validation measures [19]: accuracy (Acc), sensitivity (Sens), specificity (Spec), positive predictive value (PPV, also said precision), negative predictive value (NPV), balanced accuracy (BalAcc), and the AGF measure [24] on both the full dataset and the 100 training and test sets. In the latter case, we provided the mean values for each measure computed over the 100 repetitions and 95% confidence intervals built with the percentile method. In particular, the final best-pruned subtree derived from the application of the “1 standard error rule” in step 2 was selected as the one with the highest mean value of balanced accuracy significantly greater than 0.5 at the 0.05 significance level, as assessed through the 95% confidence intervals built on the test sets. The subtrees thus detected for each case, a, b, and c, in Section 2.2.3 were regarded as the final selected CTs to keep in the analysis. Moreover, in the multi-class classification problem of the subjects’ intentions toward lifestyle changes, balanced accuracy and AGF were computed as overall validation measures through the macro approach, i.e., by calculating the unweighted arithmetic means of balanced accuracy and AGF by class measures. Considering the nature of the considered predictors, essentially socio-demographic and behavioral variables, we considered acceptable values of AGF significantly greater than 0.5 (as assessed through the 95% confidence intervals built on the test sets). As a final assessment performed on the full dataset, we compared the performance of the final CTs with random classifiers built by randomly generating *K* labels for the classes of the CT response variables with probabilities equal to the proportions in the data. Such labels were regarded as randomly predicted classes to be compared with the true classes in the a priori classifications. To this end, the *K* labels of each random classifier were randomly permuted 10,000 times, and the above validation measures were computed using the true classes as references. The means of the validation measures and 95% confidence intervals (with the percentile method) were then computed to summarize the random classifier performance on the 10,000 random permutations. Finally, to compare the performance of a specific CT with its counterpart random classifier, a permutation test was applied for each validation measure, M, in order to verify the null hypothesis of superior performance of the random classifier RC: $H_{0}:M(RC)\geq M(CT)$ against the alternative of superior performance of the final CT: $H_{1}:M\left(RC\right)<M(CT)$.

## Appendix B

### Appendix B.1. Preliminary Data Inspection

Appendix B Table A1 and Table A2 report cross-tabulations and nonparametric test results regarding the categorical variables studied within the four age classes and between females and males, respectively. Regarding education degree, 60.14% of the subjects have at least a university degree, and the remaining 39.86% have at most a high school degree (last column in Table A1 and Table A2). However, education degrees are distributed significantly differently by age and sex. As can be seen in Table A1, there are significantly more graduates and postgraduates in the ≤30 (95.90% out of 122) and 31–50-year-old classes (79.16% out of 859) than the overall 60.14%. In the 51–64-year-old class, the graduate percentage (46.65% out of 1089) is similar to the overall graduate percentage (45.33% out of 2762), but there is a significantly greater percentage of subjects with a high school degree than overall (40.59% out of 1089 vs. 38.09% out of 2762). Similarly, among those over 65, there is a significantly higher percentage of subjects with at most a high school degree than overall (65.32% out of 692 vs. 39.86% out of 2762). Moreover, Table A2 shows no significant difference between females and males regarding primary/middle school and university degrees. However, there is a significantly higher percentage of postgraduate females (18.16% out of 1107) than males (12.57% out of 1655), and a significantly lower percentage of females with a high school degree (35.59% out of 1107) than males (39.76% out of 1655).

The job categories result in similar percentages above 20%, except for the small percentage of self-employed/freelancers (1.77% out of 2762). However, they are distributed significantly differently by age and sex. Concerning the significant positive associations between job categories and age classes (Table A1), 84.42% of those under 31 are workers/employees; in the 31–50-year-old class, 39% are workers/employees, and 39.93% are managers; in the 51–64-year-old class, 37.01% are managers, and 31.68% are directors/supervisors; finally, a very high percentage of those over 65, equal to 86.42%, is retired, as expected. Regarding the significant positive associations with sex (Table A2), it is worth noting that females are workers/employees in a higher percentage than males (40.92% out of 1107 vs. 14.98% out of 1655), while males are directors/supervisors or are retired (24.41% and 29.79% out of 1655, respectively) in a higher percentage than females (13.91% and 14.55% out of 1107, respectively). These results also align with females being younger than males overall.

Moreover, in the study set, there are many more non-smokers than smokers (87.15% vs. 12.85% out of 2762). Nonetheless, based on Table A1, smokers are in significantly higher percentages among subjects under 31 (23.77% out of 122) and in the 31–50-year-old class (16.76% out of 859) than overall, while non-smokers are 91.91% among those over 65. Moreover, female smokers make up a significantly higher percentage than males (14.91% out of 1107 vs. 11.48% out of 1655, Table A2). This latter result is also in line with females being younger than males overall.

Finally, a slight majority of subjects indicate their need for help in improving their lifestyle (53.98% yes vs. 46.02% no out of 2762). No significant difference appears in this indication across age classes (Table A1). On the other hand, females express their need for help in a significantly higher percentage than males (58.81% out of 1107 vs. 50.76% out of 1655, Table A2).

**Table A1:** Cross-tables of the (*N* = 2762) participants in this study, classified into age classes by categories of the considered categorical variables with significance tests for distributional independence and category-by-category associations ^1^.

	Age Classes (In Years Old)									
	≤30 Years		31–50 Years		51–64 Years		≥65 Years		*Total*	
Variables	*N*	%	*N*	%	*N*	%	*N*	%	*N*	%
**Sex** ^†††^										
Female	71	**58.20 *****	453	**52.74 *****	440	40.40	143	*20.66 ****	1107	40.08
Male	51	*41.80 ****	406	*47.26 ****	649	59.60	549	**79.34 *****	1655	59.92
*Total*	122	100.00	859	100.00	1089	100.00	692	100.00	2762	100.00
**Education degree** ^†††^										
Primary/middle school	0	0.00	4	*0.47 ****	23	2.11	22	**3.18 *****	49	1.77
High school	5	*4.10 ****	175	*20.37 ****	442	**40.59 ***	430	**62.14 *****	1052	38.09
University degree	65	**53.28 ***	470	**54.71 *****	508	46.65	209	*30.20 ****	1252	45.33
Post-university degree	52	**42.62 *****	210	**24.45 *****	116	*10.65 ****	31	*4.48 ****	409	14.81
*Total*	122	100.00	859	100.00	1089	100.00	692	100.00	2762	100.00
**Job category** ^†††^										
Self-employed/freelance	2	1.64	14	*1.63 **	37	**3.40 ****	14	2.02	67	2.43
Worker/employee	103	**84.42 *****	335	**39.00 *****	248	*22.77 ***	15	*2.17 ****	701	25.38
Manager	12	*9.84 ****	343	**39.93 *****	403	**37.01 *****	24	*3.47 ****	782	28.31
Director/supervisor	5	*4.10 ****	167	19.44	345	**31.68 *****	41	*5.92 ****	558	20.20
Retiree	0	*0.00 ****	0	*0.00 ****	56	*5.14 ****	598	**86.42 *****	654	23.68
*Total*	122	100.00	859	100.00	1089	100.00	692	100.00	2762	100.00
**Smoking** ^†††^										
No	93	*76.23 ****	715	*83.24 ****	963	88.43	636	**91.91 *****	2407	87.15
Yes	29	**23.77 *****	144	**16.76 *****	126	11.57	56	*8.09 ****	355	12.85
*Total*	122	100.00	859	100.00	1089	100.00	692	100.00	2762	100.00
**Need for help in improving lifestyle**										
No	55	45.08	393	45.75	496	45.55	327	47.25	1271	46.02
Yes	67	54.92	466	54.25	593	54.45	365	52.75	1491	53.98
*Total*	122	100.00	859	100.00	1089	100.00	692	100.00	2762	100.00

^1^ The Monte Carlo (MC) tests were based on 10,000 samples. Significance level codes for the Chi-square MC test for the independence of each categorical variable on age classes: ^†††^ *p* < 0.001. Significance level codes for the Z-test for the single “category-by-age-class” association (based on the standardized normal distribution of the adjusted Pearson residuals): * *p* < 0.05, ** *p* < 0.01, and *** *p* < 0.001. Percentages denoting significant positive “category-by-age-class” associations (i.e., significantly greater than the expected percentages) are in bold. Percentages denoting significant negative “category-by-age-class” associations (i.e., significantly lower than the expected percentages) are in italics.

**Table A2:** Cross-tables of the (*N* = 2762) participants in this study, classified into females and males by categories of the considered categorical variables with significance tests for distributional independence and category-by-category associations ^1^.

	Female		Male		Total	
Variables	*N*	%	*N*	%	*N*	%
**Age in class (years old)** ^†††^						
≤30 years	71	**6.41 *****	51	*3.08 ****	122	4.42
31–50 years	453	**40.92 *****	406	*24.53 ****	859	31.10
51–64 years	440	39.75	649	39.21	1089	39.43
≥65 years	143	*12.92 ****	549	**33.17 *****	692	25.05
*Total*	1107	100.00	1655	100.00	2762	100.00
**Education degree** ^†††^						
Primary/middle school	23	2.08	26	1.57	49	1.77
High school	394	*35.59 **	658	**39.76 ***	1052	38.09
University degree	489	44.17	763	46.10	1252	45.33
Post-university degree	201	**18.16 *****	208	*12.57 ****	409	14.81
*Total*	1107	100.00	1655	100.00	2762	100.00
**Job category** ^†††^						
Self-employed/freelance	31	2.80	36	2.18	67	2.43
Worker/employee	453	**40.92 *****	248	*14.98 ****	701	25.38
Manager	308	27.82	474	28.64	782	28.31
Director/supervisor	154	*13.91 ****	404	**24.41 *****	558	20.20
Retiree	161	*14.55 ****	493	**29.79 *****	654	23.68
*Total*	1107	100.00	1655	100.00	2762	100.00
**Smoking** ^††^						
No	942	*85.09 ***	1465	**88.52 ****	2407	87.15
Yes	165	**14.91 ****	190	*11.48 ***	355	12.85
*Total*	1107	100.00	1655	100.00	2762	100.00
**Need for help in improving lifestyle** ^†††^						
No	456	*41.19 ****	815	**49.24 *****	1271	46.02
Yes	651	**58.81 *****	840	*50.76 ****	1491	53.98
*Total*	1107	100.00	1655	100.00	2762	100.00

^1^ The Monte Carlo (MC) tests were based on 10,000 samples. Significance level codes for the Chi-square MC test for the independence of each categorical variable on sex: ^††^ *p* < 0.01, and ^†††^ *p* < 0.001. Significance level codes for the Z-test for the single “category-by-sex” association (based on the standardized normal distribution of the adjusted Pearson residuals): * *p* < 0.05, ** *p* < 0.01, and *** *p* < 0.001. Percentages denoting significant positive “category-by-sex” associations (i.e., significantly greater than the expected percentages) are in bold. Percentages denoting significant negative “category-by-sex” associations (i.e., significantly lower than the expected percentages) are in italics.

Appendix B Table A3 and Table A4 include summary statistics and nonparametric test results for the ordinal and quantitative variables studied within the four age classes and between females and males, respectively. Table A3 shows significant differences across age classes concerning weight, waist circumference, BMI, the total MET volume of physical activity, the three perceived somatic symptom scales, and the job performance quality scale. Similar test results are reported for sex (Table A4), except for height, which is significantly lower in females than in males, as expected, and the job performance quality scale, for which no significant difference was detected between females and males. Moreover, no significant differences were noted regarding the quality of nutrition across age classes or between females and males for which the median AHA diet score is equal to 2 (±1) in all the considered cases.

**Table A3:** Summary statistics (median ± MAD) of the studied quantitative and ordinal variables within age classes and two-tailed Monte Carlo Kruskal–Wallis and median significance tests ^1^.

	Age Classes (In Years Old)				
Variables	≤30 Years	31–50 Years	51–64 Years	≥65 Years	*Total*
**Anthropometrics**					
Weight (kg) ***^,†††^	62.50 ± 8.50	69.00 ± 11.00	74.00 ± 10.00	74.5 ± 8.00	72.00 ± 10.00
Height (cm) *	172.00 ± 8.00	171.00 ± 7.00	173.00 ± 6.00	172.50 ± 5.50	172.00 ± 6.00
Waist circumference (cm) ***^,†††^	76.50 ± 10.50	84.00 ± 11.00	90.00 ± 9.00	96.00 ± 7.00	90.00 ± 10.00
Body Mass Index—BMI (kg/m^2^) ***^,†††^	21.87 ± 1.89	23.33 ± 2.14	24.14 ± 2.10	24.82 ± 1.93	24.02 ± 2.17
**Lifestyle variables**					
Hours/night of sleep ^2^	7.00 ± 1.00	7.00 ± 1.00	7.00 ± 1.00	7.00 ± 1.00	7.00 ± 1.00
Total METs (minutes/week) **^,†††^	1170.85 ± 770.95	777.00 ± 610.80	975.00 ± 645.00	850.50 ± 553.50	882.50 ± 642.50
AHA diet score	2.00 ± 1.00	2.00 ± 1.00	2.00 ± 1.00	2.00 ± 1.00	2.00 ± 1.00
**Perceived somatic symptom scales**					
Short 4SQ ***^,†††^	9.00 ± 6.00	5.00 ± 5.00	4.00 ± 4.00	2.00 ± 2.00	4.00 ± 4.00
Fatigue ***^,†††^	6.00 ± 2.00	5.00 ± 2.00	3.00 ± 2.00	2.00 ± 2.00	3.00 ± 2.00
Stress ***^,†††^	6.00 ± 2.00	5.00 ± 2.00	4.00 ± 2.00	1.00 ± 1.00	3.00 ± 2.00
**Perceived quality scales**					
Sleep	7.00 ± 1.00	7.00 ± 1.00	7.00 ± 1.00	7.00 ± 1.00	7.00 ± 1.00
Health	7.00 ± 1.00	7.00 ± 1.00	7.00 ± 1.00	7.00 ± 1.00	7.00 ± 1.00
Job performance ***^,†††^	8.00 ± 1.00	8.00 ± 1.00	8.00 ± 1.00	5.00 ± 5.00	8.00 ± 1.00

^1^ The Monte Carlo tests were based on 10,000 samples. Two-tailed Kruskal–Wallis test: $H_{0}:\tau_{1}=\tau_{2}=\tau_{3}=\tau_{4}$ against $H_{1}:\tau_{j}\neq \tau_{r}$ for at least one j≠r, with $\tau_{j}$ the effect of the *j*-th age class on variable *X*. Significance level: * *p* < 0.05, ** *p* < 0.01, and *** *p* < 0.001. Two-tailed median test: $H_{0}:\theta_{1}=\theta_{2}=\theta_{3}=\theta_{4}$ against $H_{1}:\theta_{j}\neq \theta_{r}$ for at least one j≠r, with $\theta_{j}$ the median of variable *X* in the *j*-th age class. Significance level: ^†††^ *p* < 0.001. ^2^ There are 24 missing values.

In addition to the above, it is worth noting that the youngest subjects were characterized, as expected, by the best anthropometric profile (i.e., waist circumference and BMI tendentially lower than in the other age classes) and with a higher total MET volume of physical activity (Table A3). Nevertheless, the youngest subjects reported the worst median scores on the short 4SQ, fatigue, and stress somatic symptom scales (Table A3), as well as the females compared to the males (Table A4). Finally, no significant differences were detected in the perceived quality scales of sleep and health across age classes and between females and males. In all cases, the median scores of the two scales equal 7 (±1), thus denoting that subjects’ perception of sleep and health quality is good overall. Regarding the job performance quality scale, as already mentioned, females and males do not report significant differences; the median score of this scale equals 8 (±1) for both, which indicates a high level of the perceived quality of their job performance (Table A4). In contrast, a lower median score on this scale was observed for subjects over 65 (5 ± 5) than for those in the other age classes (8 ± 1).

**Table A4:** Summary statistics (median ± MAD) of the studied quantitative and ordinal variables within sex and two-tailed Monte Carlo Kruskal–Wallis and median significance tests ^1^.

Variables	Female	Male	*Total*
**Anthropometrics**			
Weight (kg) ***^,†††^	60.00 ± 6.00	78.00 ± 7.00	72.00 ± 10.00
Height (cm) ***^,†††^	165.00 ± 5.00	177.00 ± 5.00	172.00 ± 6.00
Waist circumference (cm) ***^,†††^	80.00 ± 10.00	95.00 ± 7.00	90.00 ± 10.00
Body Mass Index—BMI (kg/m^2^) ***^,†††^	22.15 ± 2.08	24.90 ± 1.86	24.02 ± 2.17
**Lifestyle variables**			
Hours/night of sleep ^2^	7.00 ± 1.00	7.00 ± 1.00	7.00 ± 1.00
Total METs (minutes/week) ***^,††^	786.00 ± 594.00	975.00 ± 655.00	882.50 ± 642.50
AHA diet score	2.00 ± 1.00	2.00 ± 1.00	2.00 ± 1.00
**Perceived somatic symptom scales**			
Short 4SQ ***^,†††^	6.00 ± 5.00	3.00 ± 3.00	4.00 ± 4.00
Fatigue ***^,†††^	5.00 ± 3.00	3.00 ± 2.00	3.00 ± 2.00
Stress ***^,†††^	5.00 ± 3.00	3.00 ± 2.00	3.00 ± 2.00
**Perceived quality scales**			
Sleep	7.00 ± 1.00	7.00 ± 1.00	7.00 ± 1.00
Health	7.00 ± 1.00	7.00 ± 1.00	7.00 ± 1.00
Job performance	8.00 ± 1.00	8.00 ± 1.00	8.00 ± 1.00

^1^ The Monte Carlo tests were based on 10,000 samples. Two-tailed Kruskal–Wallis test: $H_{0}:\tau_{1}=\tau_{2}$ against $H_{1}:\tau_{1}\neq \tau_{2}$, with $\tau_{j}$ the effect of the *j*-th sex on variable *X*. Significance level: *** *p* < 0.001. Two-tailed median test: $H_{0}:\theta_{1}=\theta_{2}$ against $H_{1}:\theta_{1}\neq \theta_{2}$, with $\theta_{j}$ the median of variable *X* in the *j*-th sex. Significance level: ^††^ *p* < 0.01, and ^†††^ *p* < 0.001. ^2^ There are 24 missing values.

### Appendix B.2. Classification Trees

**Table A5:** Collection of tables with frequency and percentage distributions of the categorized variables involved in the classification tree construction: waist circumference, BMI, lifestyle variables, perceived somatic symptom scales, and perceived quality scales ^1^.

WC ^2^	count	%	BMI	count	%	Hours/night of sleep	count	%
no risk	1414	51.19%	<25	1721	62.31%	1–5	376	13.61%
medium risk	623	22.56%	[25, 30)	863	31.25%	6–7	1829	66.22%
high risk	725	26.25%	≥30	178	6.44%	8–12	533	19.30%
	2762	100.00%		2762	100.00%	missing	24	0.87%
							2762	100.00%
**Total METs**	**count**	**%**	**AHA score**	**count**	**%**	**Short 4SQ**	**count**	**%**
<600	1042	37.73%	0–1	574	20.78%	0–3	1332	48.23%
≥600	1720	62.27%	2–3	1858	67.27%	4–8	659	23.86%
	2762	100.00%	4–5	330	11.95%	9–36	771	27.91%
				2762	100.00%		2762	100.00%
**Fatigue**	**count**	**%**	**Stress**	**count**	**%**	**Sleep q**	**count**	**%**
0–3	1451	52.53%	0–3	1424	51.56%	0–3	251	9.09%
4–6	682	24.69%	4–6	700	25.34%	4–6	1030	37.29%
7–10	629	22.77%	7–10	638	23.10%	7–10	1481	53.62%
	2762	100.00%		2762	100.00%		2762	100.00%
**Health q**	**count**	**%**	**Job perf q**	**count**	**%**			
0–3	69	2.50%	0–3	365	13.22%			
4–6	822	29.76%	4–6	236	8.54%			
7–10	1871	67.74%	7–10	2161	78.24%			
	2762	100.00%		2762	100.00%			

^1^ Abbreviations: WC = waist circumference; BMI = Body Mass Index; Sleep q = perceived sleep quality scale; Health q = perceived health quality scale; Job perf q = perceived quality scale of job performance. ^2^ Categorization of waist circumference: (1) no risk: males with WC ≤ 94 cm and females with WC ≤ 80 cm; (2) medium risk: males with 94 < WC < 102 cm and females with 80 < WC < 88 cm; (3) high risk: males with WC ≥ 102 cm and females with WC ≥ 88 cm.

**Table A6:** Construction of the classification trees: pre-processing step and assessment of variable importance on the pruned classification trees ^1^.

	Pre-Processing ^2^				After Pruning			
	CT in Cases a and b		CT in Cases c and d		Overall Variable Importance ^2^			
Predictors	Freq Ratio	% Unique	Freq Ratio	% Unique	VI CT-a	VI CT-b	VI CT-c	VI CT-d
sex	1.495	0.072	1.432	0.081	0.371	0.695	**8.423**	0
age	1.268	0.145	1.216	0.162	0.413	0.496	**33.300**	**4.771**
educ degr	1.190	0.145	1.246	0.162	0	0	0	**4.061**
job cat	1.116	0.181	1.085	0.203	0.397	**8.808**	2.778	0.702
smoking	6.780	0.072	6.558	0.081	**15.075**	0	0	0.631
help	1.173	0.072	1.444	0.081	0	**44.290**	0	**14.626**
WC	1.950	0.109	2.011	0.122	0.377	0	0	0
BMI	1.994	0.109	2.024	0.122	0.377	0	1.151	1.342
h/n sleep	3.432	0.109	3.600	0.122	0.753	0	0	0
tot METs	1.651	0.072	1.699	0.081	**24.280**	0	0	0.610
AHA	3.237	0.109	3.233	0.122	0.742	0	0	0
short 4SQ	1.728	0.109	1.589	0.122	0.472	0.339	2.433	0
fatigue	2.128	0.109	1.950	0.122	2.197	0.715	2.555	0.668
stress	2.034	0.109	1.869	0.122	**17.264**	0.725	**14.183**	0.677
sleep q	1.438	0.109	1.425	0.122	**35.725**	0	1.233	0
health q	2.276	0.109	2.307	0.122	1.194	0	0	0.777
job perf q	5.921	0.109	6.958	0.122	0.363	0.419	1.503	0
econ inv	1.581	0.145	2.084	0.162	0	**43.513**	**18.808**	**2.821**
intentions ^3^	2.280	0.181	2.429	0.203	----	0	**13.633**	**68.314**

^1^ All the considered quantitative/ordinal predictors were categorized into classes (see Table A1 and Table A2 for age and Table A5). Abbreviations: educ degr = education degree; job cat = job category; smoking = smoking habit; help = need for help in improving lifestyle; WC = waist circumference; BMI = Body Mass Index; h/n sleep = hours/night of sleep; tot METs = total METs physical activity volume; AHA = AHA diet score; sleep q = perceived sleep quality scale; health q = perceived health quality scale; job perf q = perceived quality scale of job performance; econ inv = economic willingness to invest in target actions to improve lifestyles; intentions = subjects’ intentions toward lifestyle changes; VI = variable importance; CT-x = classification tree built for case x described in Section 2.2.3 and Section 3.3. ^2^ The two pre-processing indices, “freq ratio” (frequency ratio) and “% unique” (percentage of unique categories), along with the VI measure reported in this table, were implemented in the R “caret” library [30]. The VI measure was rescaled to add 100 for each CT. The VI values of the most important predictors in the CTs selected by pruning are in bold and highlighted in light green. ^3^ “Subjects’ intentions toward lifestyle changes” is the response variable in CT of case a. Accordingly, its importance as a predictor was not assessed and then not reported in the column “VI CT-a”.
